# The *Coptotermes gestroi* aldo–keto reductase: a multipurpose enzyme for biorefinery applications

**DOI:** 10.1186/s13068-016-0688-6

**Published:** 2017-01-03

**Authors:** Robson Tramontina, João Paulo L. Franco Cairo, Marcelo V. Liberato, Fernanda Mandelli, Amanda Sousa, Samantha Santos, Sarita Cândida Rabelo, Bruna Campos, Jaciane Ienczak, Roberto Ruller, André R. L. Damásio, Fabio Marcio Squina

**Affiliations:** 1 Laboratório Nacional de Ciência e Tecnologia do Bioetanol (CTBE), Centro Nacional de Pesquisa em Energia e Materiais (CNPEM), Rua Giuseppe Máximo Scolfaro, no 10000 Campinas, SP Brazil; 2 Programa de Pós Graduação em Biociências e Tecnologia de Produtos Bioativos (BTPB)-Instituto de Biologia-CP 6109, Universidade Estadual de Campinas-UNICAMP, 13083-970 Campinas, SP Brazil; 3 Brazilian Biosciences National Laboratory (LNBio), from the Brazilian Center for Research in Energy and Materials (CNPEM), Campinas, Brazil; 4 Department of Biochemistry and Tissue Biology, Institute of Biology, University of Campinas (UNICAMP), Campinas, SP Brazil

**Keywords:** *Coptotermes gestroi*, Aldo–keto reductase, Bioethanol, Detoxification, Reactive oxygen species

## Abstract

**Background:**

In nature, termites can be considered as a model biological system for biofuel research based on their remarkable efficiency for lignocellulosic biomass conversion. Redox enzymes are of interest in second-generation ethanol production because they promote synergic enzymatic activity with classical hydrolases for lignocellulose saccharification and inactivate fermentation inhibitory compounds produced after lignocellulose pretreatment steps.

**Results:**

In the present study, the biochemical and structural characteristics of the *Coptotermes gestroi* aldo–keto reductase (*Cg*AKR-1) were comprehensively investigated. *Cg*AKR-1 displayed major structural differences compared with others AKRs, including the differences in the amino acid composition of the substrate-binding site, providing basis for classification as a founding member of a new AKR subfamily (family AKR1 I). Immunolocalization assays with anti-*Cg*AKR-1 antibodies resulted in strong fluorescence in the salivary gland, proventriculus, and foregut. *Cg*AKR-1 supplementation caused a 32% reduction in phenolic aldehydes, such as furfural, which act as fermentation inhibitors of hemicellulosic hydrolysates, and improved ethanol fermentation by the xylose-fermenting yeast *Scheffersomyces stipitis* by 45%. We observed synergistic enzymatic interactions between *Cg*AKR-1 and commercial cellulosic cocktail for sugarcane bagasse saccharification, with a maximum synergism degree of 2.17 for sugar release. Our data indicated that additive enzymatic activity could be mediated by reactive oxygen species because *Cg*AKR-1 could produce hydrogen peroxide.

**Conclusion:**

In summary, we identified the founding member of an AKRI subfamily with a potential role in the termite digestome. *Cg*AKR-1 was found to be a multipurpose enzyme with potential biotechnological applications. The present work provided a basis for the development and application of integrative and multipurpose enzymes in the bioethanol production chain.

**Electronic supplementary material:**

The online version of this article (doi:10.1186/s13068-016-0688-6) contains supplementary material, which is available to authorized users.

## Background

Lignocellulose is a recalcitrant matrix composed of cellulose, hemicelluloses, and lignin, and a complex set of enzymes are required for the efficient conversion of these plant cell wall polymers into fermentable sugars [1]. Termites can degrade almost 90% of consumed plant biomass and can provide an excellent biological system for studying the biochemical depolymerization of lignocellulosic biomass [2, 3]. The gut of *Coptotermes gestroi* (Rhinotermitidae) and other termites has specialized adaptations to digest lignocellulosic diets [4]. In the termite gut, carbohydrate-active enzymes (CAZymes), such as cellulases and hemicellulases, are secreted (i.e., both symbiotic and endogenous enzymes). Additionally, a set of pro-oxidant, antioxidant, and detoxification enzymes (PADs) is also present [5–7]. Among the PADs found in termites, superoxide dismutase (SOD), catalases (CATs), glutathione S-transferase (GST), and aldo–keto reductases (AKRs) have been studied in detail because the transcription of mRNAs encoding these enzymes is upregulated in response to lignocellulose degradation [8].

One of the major bottlenecks for second-generation ethanol production is the toxic metabolites produced after lignocellulose pretreatment [9]. Different lignocellulose pretreatments, such as diluted acids, are required to minimize biomass recalcitrance, alter the biomass structure, and enhance the enzymatic degradation of lignocellulose [10]. However, during lignocellulosic biomass pretreatment, several chemical by-products are generated, which inhibit fermentative microorganisms and lignocellulolytic enzymes [11]. These chemicals include aldehydes, aliphatic acids, furan derivatives, and phenolic compounds, such as hydroxybenzoic acid, furfural, and hydroxymethylfurfural (HMF) [9, 12, 13]. For example, the presence of furfural can strongly inhibit the growth of many yeast strains by cell wall and membrane damage, enzymatic activity inhibition, DNA damage, and protein and RNA synthesis [13, 14].

Physicochemical and biological strategies are being developed to minimize the effects of these inhibitors on enzymatic and microbial activity for second-generation ethanol [11]. Recently, Liu et al. [15] highlighted the importance of developing an easy-to-handle in situ detoxification method combined with a fermentation process in order to produce second-generation ethanol from low-cost lignocellulosic biomass. However, with the exception of microbial laccases and peroxidases, such products have not been reported frequently [10]. Therefore, PADs and related enzymes may have many applications in the detoxification of lignocellulosic hydrolysates [8, 11, 16, 17].

Studies of the oxidoreductive mechanisms that can improve lignocellulose biomass saccharification have shown that laccases, peroxidases, and other auxiliary redox activities enzymes can enhance biomass hydrolysis by acting on the recalcitrance of woody materials by direct or indirect oxidation of holocellulose [18–20].

The involvement of redox enzymes in lignocellulose modification and degradation in the termite digestome has not been fully elucidated [2, 7, 21–23]. Previous studies suggested that enzymes related to redox reactions and detoxifying metabolism may improve the ability of termites to digest a lignocellulosic diet. For example, hydrogen peroxide and reduced iron were found in the guts of *Coptotermes formosanus* and *Zootermopsis nevadensis*, respectively, in acidic pH conditions [2, 22, 24]. These results suggested that highly reactive radicals were generated in the termite gut [24, 25].

Franco Cairo et al. [26] showed that AKR transcripts from the termite *Coptotermes gestroi* were abundant in worker castes (responsible for colony feeding [27].) The AKR superfamily of proteins is known to catalyze the NAD[P]H-dependent reduction of various carbonyl-containing compounds to their corresponding alcohols, and systematic nomenclature for the AKR superfamily has been in place since 1996 (www.med.upenn.edu/akr) [28]. Moreover, AKRs are involved in several metabolic reactions in different organisms, including carbohydrate degradation, xenobiotic detoxification, degradation of β-aryl ethers in lignin, and various industrial and clinical applications [29–31].

In this work, we describe the AKR from the termite *C. gestroi* (*Cg*AKR-1), a founding member of a new AKR subfamily of potential biotechnological interest. To the best of our knowledge, this is the first report in the literature to describe the use of AKR for detoxification of fermentation inhibitors during C5 ethanol fermentation. Furthermore, this work provides a basis for studies of the synergistic enzymatic interactions of AKRs with cellulases and the use of multipurpose enzymes for bioprocess integration aiming to improve lignocellulosic biorefinery performance.

## Results and discussion

### CgAKR-1 was a founding member of a new AKR1I subfamily

Recently, several research groups have investigated the termite digestome, hypothesizing that auxiliary redox mechanisms may be involved in lignocellulose degradation [5, 26, 27, 32–34]. Among these studies, Scharf and Sethi [27] reported that an AKR acted synergically with termite and symbiotic GHs during pine wood hydrolysis. *AKR* transcripts were recently found to be abundantly expressed in *C. gestroi* worker castes when consuming a diet based on pinewood, suggesting that some AKRs from *C. gestroi* are highly expressed in response to lignocellulosic material. However, few details of the biochemical and structural properties of termite AKR have been reported [27].

**Fig. 1:**
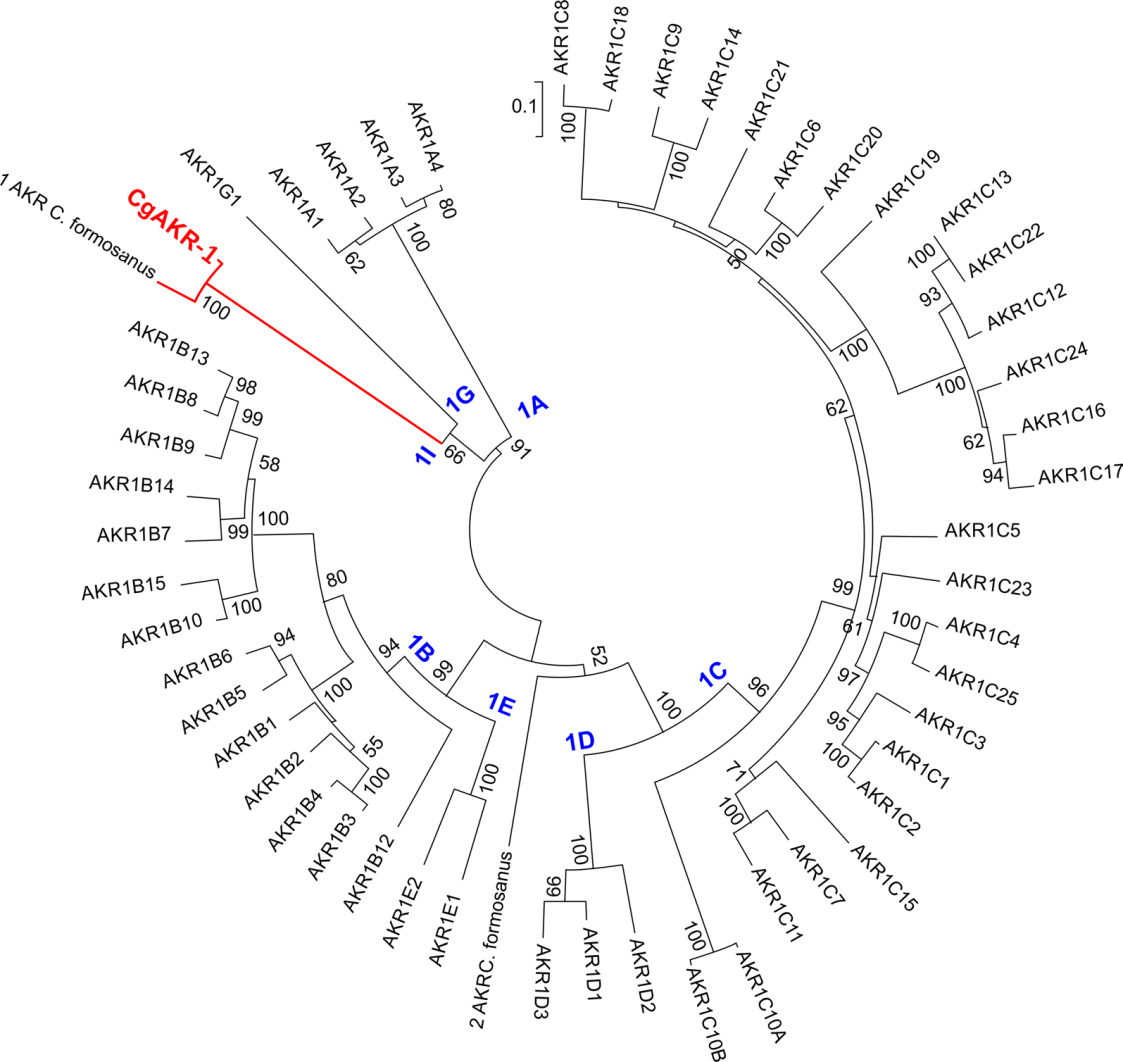
Phylogenetic tree of members of the AKR superfamily. Amino acid sequences (AKR1 family) were found in the AKR database (https://www.med.upenn.edu/akr/), and *Cg*AKR-1 and a related termite AKR sequence (AGM32584.1) were added. The dendrogram was generated as described by Hyndman [38]. The tree was constructed with the neighbor-joining method implemented in MEGA6.0 using 1000 bootstraps. The evolutionary distances were computed using the JTT matrix-based method and are presented as the number of amino acid substitutions per site. Evolutionary analyses were conducted in MEGA6

The predicted open reading frame (ORF) of *CGAKR1*, based on genomic data, contained 334 amino acids (GenBank accession number: KU686221). The domain architecture evaluation performed by comparison of the *Cg*AKR-1 protein sequence with the PFAM database indicated the presence of a conserved domain from the AKR superfamily. Moreover, a comparison of the predicted protein *Cg*AKR-1 with the NCBI database indicated higher similarity to a protein from *C. formosanus* (97% identity, accession number AGM32584.1 [1AKR]). According to data from the AKR superfamily homepage, AKRs are found in both prokaryotes and eukaryotes and are distributed among 16 families [35]. A phylogenetic tree was constructed using the amino acid sequence from all 16 AKR family members, in which *Cg*AKR-1 was located in family 1 (Additional file 1: Figure S1). Subsequently, another phylogenetic tree was constructed using the amino acid sequence of AKRs from family 1 only, and *Cg*AKR-1 was clustered in an unaffiliated clade and classified as a novel AKR subfamily (Fig. 1). According to the nomenclature specifications, *Cg*AKR-1 was a founding member of the new subfamily “AKR1 I” [35]. The most related and well-characterized family members in this database are human AKR (AKR1A1) and nematode AKR from *Caenorhabditis elegans* (AKR1G1) [35]. Generally, members of the AKR1 family have broad specificity for aldehydes, are cytosolic and monomeric proteins, and interact strongly with NADPH as a cofactor [30, 35].

The gene-encoding *Cg*AKR-1 was successfully cloned in *Escherichia coli* ArcticExpress DE3 competent cells, and the soluble enzyme was purified by affinity and size exclusion chromatography steps with high enzyme yield (25 mg/L of cell culture; see Additional file 1: Figure S2).

### *Cg*AKR-1 was primarily localized in the termite foregut region

**Fig. 2:**
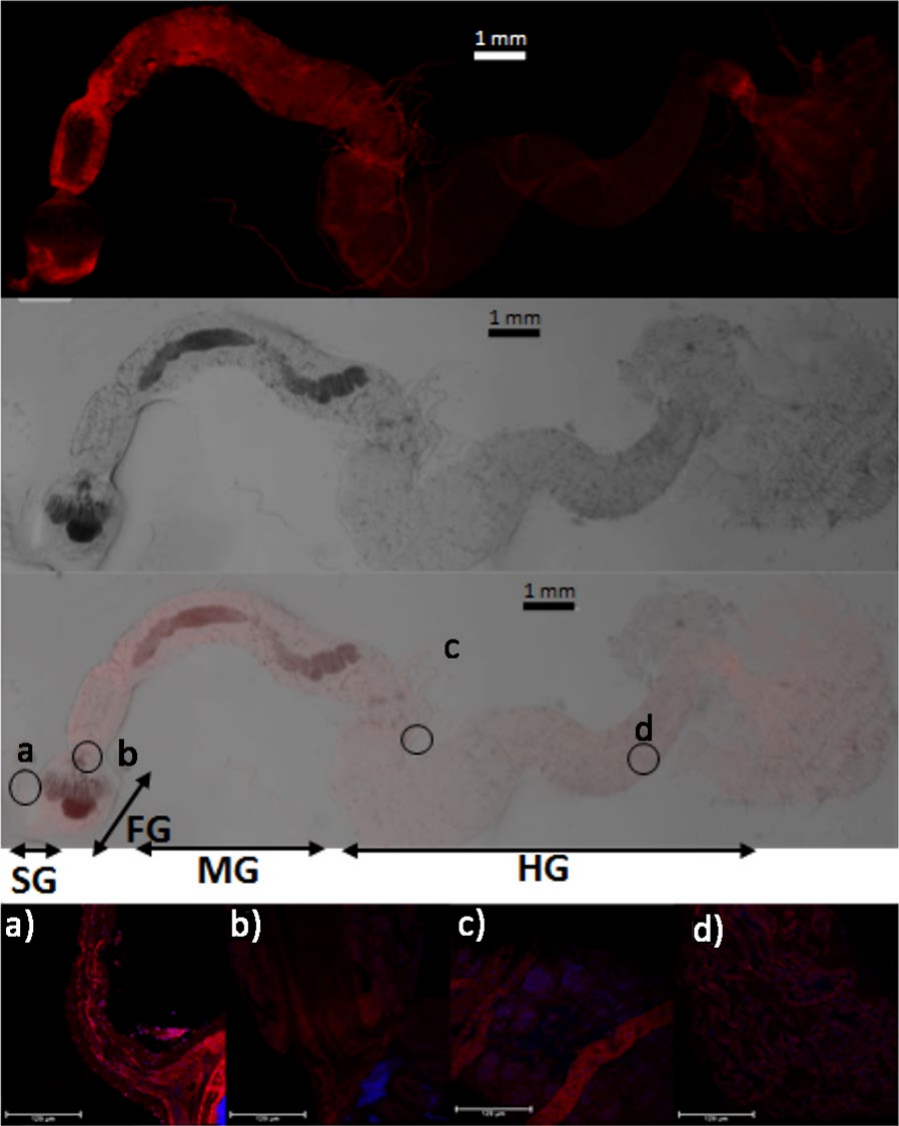
Immunolocalization of *Cg*AKR-1 in *C. gestroi* gut tissues. Gut tissues were incubated with primary anti-*Cg*AKR-1 antibodies and AlexaFluor 568 secondary antibodies and observed under a Leica DMI 6000 microscope. *Red fluorescence* indicates anti-*Cg*AKR-1 antibody binding; *gray* represents the gut visualization under white light; and *blue fluorescence* represents the nucleus in all cells using ProLong Antifade Reagent for Fixed Cells. Images from the* red*,* blue*, and* white* channels were recorded independently and digitally overlaid to produce a* final image*. Whole gut: salivary glands (SG); foregut (FG); midgut (MG); hindgut (HG). The letters indicate multiple layer image analysis:  *a*) foregut, *b*) midgut, *c*) malphigian tubes *d*) hindgut. The scale bar corresponds to 124 μm length. Further details of immunolocalization of *Cg*AKR-1 are presented in the Additional file 1

The detection of nonsymbiotic phenoloxidase activities in termites has already been described, supporting the oxidative degradation of lignin and cellulose in the gut of termites [3, 33]. However, elucidation of the redox mechanisms in the termite digestome is necessary. Accordingly, we next investigated the immunolocalization of *Cg*AKR-1 in the *C. gestroi* gut for the first time (Fig. 2).

The interaction between anti-*Cg*AKR-1 antibodies and target proteins in the gut of *C. gestroi* was investigated by immunolocalization. After incubation with both primary and secondary antibodies, the gut tissue showed strong fluorescence mainly located in the salivary gland, proventricle, and foregut (Fig. 2a). In contrast, there was nearly a completely lack of fluorescence in the hindgut (Fig. 2d). Strong fluorescence was also observed in the midgut (Fig. 2b) and the junction of the foregut and midgut. The foregut lumen of lower termites has high oxygen potential and harbors most digestive enzymes [2], such as *C. gestroi* endoglucanase (see Additional file 1: Figure S5). Thus, *Cg*AKR-1 would be expected to be expressed in this gut section. In addition, *Cg*AKR-1 was also detected in the malpighian tubules attached in the midgut (Fig. 2c). To the best of our knowledge, some PAD enzymes, such as cytochrome oxidase [36], a candidate enzyme involved in enzymatic detoxification and lignin degradation in termites [34, 37], are expressed in the malpighian tubules.

### *Cg*AKR-1 was active against yeast fermentation inhibitor compounds

**Fig. 3:**
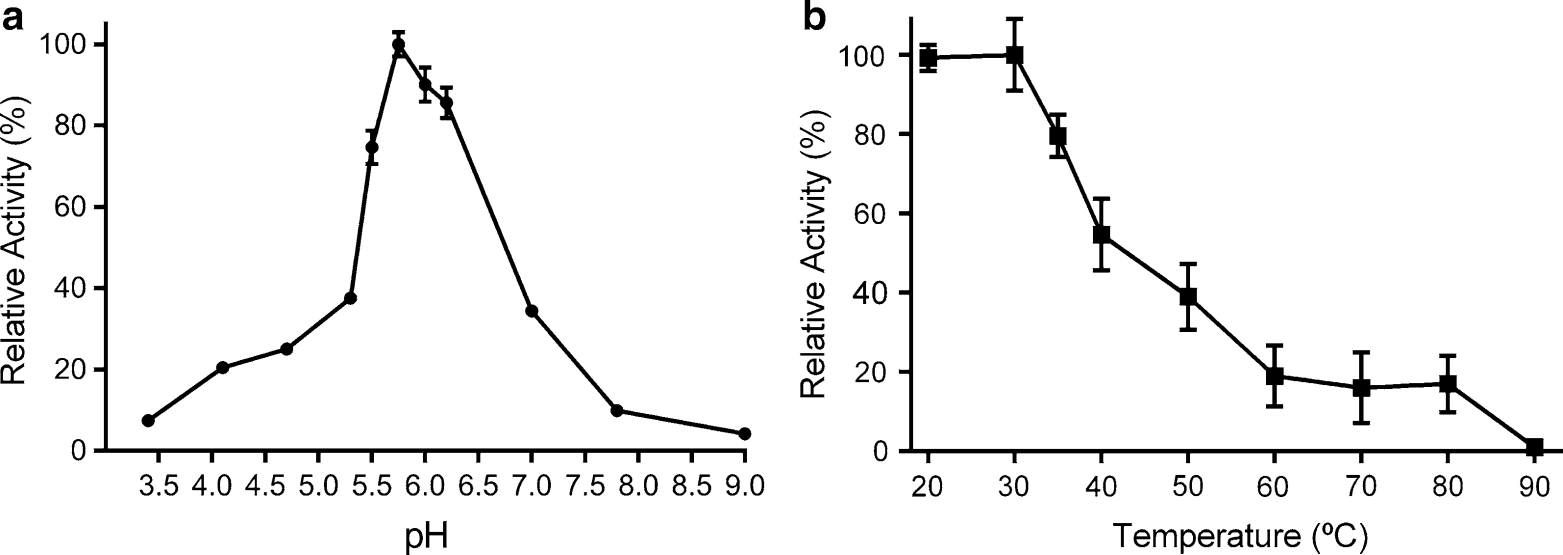
Biochemical properties of *Cg*AKR-1. **a** Effects of pH on *Cg*AKR-1 activity. Phosphate buffer (pH 3.0–8.0) and Tris buffer (pH 8.5–9.0) were used at 100 mM at 30 °C. The results were expressed as relative activity (%) in relation to optimal pH of 5.7. **b** *Cg*AKR-1 thermostability. The enzyme was incubated at pH 5.7 for 30 min at different temperatures. After incubation, the standard enzyme assay was performed, and the results were expressed as relative activity (%) in relation to the optimal temperature of 30 °C

Recombinant *Cg*AKR-1 showed high affinity for the standard substrate 2-nitrobenzaldehyde (NBZ), with a V_max_ of 2.34 U/mg., K_m_ of 0.15 mmol/L, and K_cat_ of 7.5 s^−1^. In addition, the K_d_ for cofactor NADPH was 0.06 mmol/L. No activity was observed when NADH was used as a cofactor (data not shown). The specific activity of *Cg*AKR-1 on NBZ was 1.53 μmol/mg/min under optimal conditions at pH 5.7 (Fig. 3a) and 30 °C (Fig. 3b). This activity level was higher than that reported for *Saccharomyces cerevisiae* AKR (0.34 μmol/mg/min) [38] and lower than that reported for human AKR1A1 under optimal conditions (2.47 μmol/mg/min) [37].

The optimal pH of the enzyme was within the same range found in the gut of lower termites and was similar to the pH reported for rabbit AKR (pH 5.6) [38]. Notably, however, aldehyde reductases from animals and fungi typically have an optimal pH at or near neutral [28, 31, 39, 40]. Furthermore, *Cg*AKR-1 was stable in the range of 20–35 °C and maintained residual activity (about 30%) at temperatures from 50 to 80 °C; no activity was observed at 90 °C (Fig. 3b).

Table 1 shows the specificity of *Cg*AKR-1 for different substrates, as measured by the oxidation of NADPH. The high specificity of *Cg*AKR-1 for 2-nitrobenzaldehyde has also been reported for various AKRs [39]. In general, the enzyme had high activity for aromatic and aliphatic aldehydes. No reductase activity was detected for vanillin, aldose sugars, propanone, and polysaccharides (data not shown). According to our data, *Cg*AKR-1 was active on several chemicals found in hemicellulosic hydrolysates from sugarcane bagasse (SCB), such as syringaldehyde, hydroxybenzaldehyde, HMF, and furfural, which can inhibit yeast fermentation [11]. The activity of an intestinal AKR on these aromatic aldehyde molecules could prevent electrophilic injury caused by these compounds and would be consistent with the fact that a similar AKR was induced by a lignin-rich diet in *Reticulitermes flavipes* [7, 28].

**Table 1:** *Cg*AKR-1 substrate specificity

Substrate (2 mmol/L)	Relative activity (%)	Structure
2-Nitrobenzaldehyde	100	
5-Hydroxymethylfurfural	116	
Furfural	112	
4-Hydroxybenzaldehyde	61	
Syringaldehyde	72	
Glutaraldehyde	98	
Acetaldehyde	59	

Specific activity was determined as µmol of NADPH oxidized per min per mg protein. Relative activities of *Cg*AKR-1 using different substrates are presented as the percentage of *Cg*AKR-1 activity on 2-nitrobenzaldehyde

**Fig. 4:**
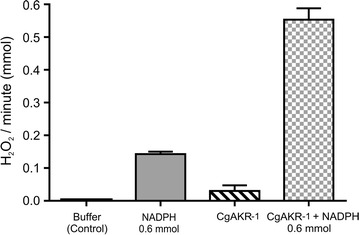
H_2_O_2_ production per minute by *Cg*AKR-1 and its cofactor NADPH, as measured with Amplex Red. All reactions were performed in triplicate in 100 mM phosphate buffer (pH 5.7) at 37 °C

AKRs are able to generate H_2_O_2_ and other reactive oxygen species (ROS) via NADPH oxidation [41, 42]. We performed in vitro assays to quantify H_2_O_2_ production by *Cg*AKR-1 because H_2_O_2_ can contribute to lignocellulose deconstruction [43–45]. Amplex Red assays revealed that *Cg*AKR-1 generated 0.55 mmol of H_2_O_2_ per minute in the presence of 0.6 mmol NADPH (Fig. 4). The H_2_O_2_ generated from *Cg*AKR-1 was able to initiate the Fenton reaction in the presence of Fe^2+^, generating the ^•^OH radical, a powerful oxidant that can be utilized in lignocellulose degradation [45] (detected by a peroxynitrite sensor [HPF]; see Additional file 1: Figure S7).

Thus, we concluded that in addition to its ability to reduce aldehydes, *Cg*AKR-1 had an NADPH oxidase-like function and could generate ROS, such as H_2_O_2_, during in vitro assays. In the presence of reducing agents, such as Fe^2+^ (present in termite guts), the hydroxyl radical could also be produced through the action of *Cg*AKR-1.

### Loop B of *Cg*AKR-1 was longer than that of other AKRs

**Fig. 5:**
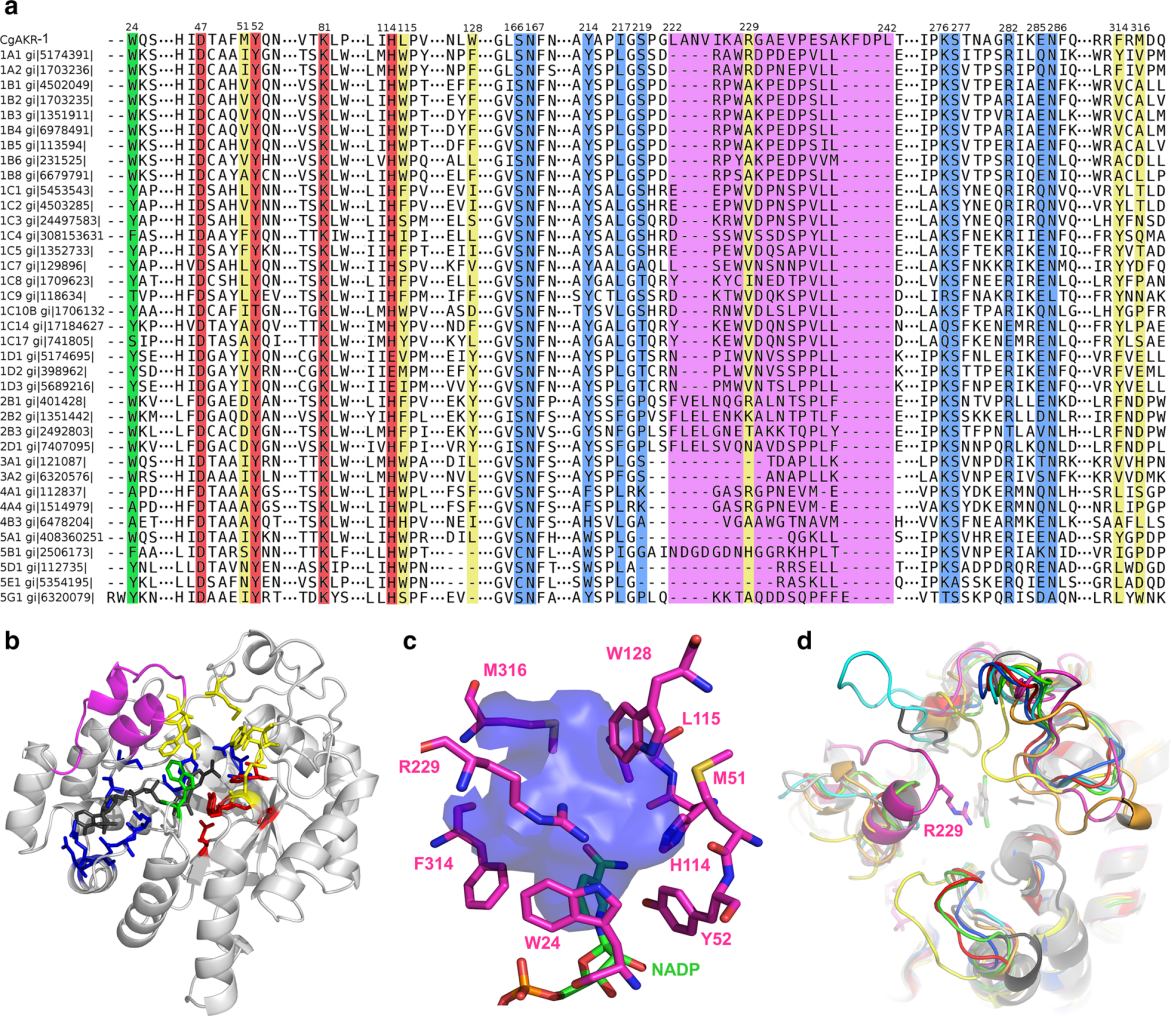
Sequence comparison and structural assignment. **a** Multiple sequence alignment of *Cg*AKR with members of the most similar related families AKR1–5. Despite the low identity among *Cg*AKR-1 and other AKR enzymes, the catalytic residues (*red*) and the residues that interacted with the cofactor (*blue*) had a high degree of similarity. The residues that surrounded the substrate (*yellow*) were more diverse between each family, reflecting the specificity for different substrates. The position that interacted with both the substrate and cofactor is shown in *green*. One of the main differences between *Cg*AKR-1 and the other enzymes was the presence of a longer loop, known as loop B (*magenta*), located in the substrate-binding site. **b** Positioning of the important amino acids in the *Cg*AKR-1 crystallographic structure (the colors in **b** are the same as those in **a**). **c** Substrate-binding site. The cavity in the *Cg*AKR-1 structure, represented here as a *blue* surface, was determined to be the substrate-binding site by comparison with other substrate-complexed AKRs. Therefore, the pocket was mainly delimited by the cofactor (NADP) and nine amino acids with different properties, including R229 from loop B (observed only in *Cg*AKR-1). **d** Superposition of *Cg*AKR-1 with other AKRs from different families: 1A1 complexed with 3,5-dichlorosalicylic acid (*gray arrow*) in gray (PDBid 3cv7), 1B in *blue* (PDBid 4hbk), 1C in *green* (PDBid 1ihi), 1D in *red* (PDBid 3buv), 2B in *orange* (PDBid 1mi3), 4A in *yellow* (PDBid 1zgd), and 5C in *black* (PDBid 1hw6). The loops comprised the entrance to the substrate-binding site. Despite the discernible variations in size and position, *Cg*AKR-1 (*magenta*) had one loop (loop B, indicated with a *magenta arrow*) that was longer than the others. This loop also contained an arginine (R229) that may interact with the substrate

The three-dimensional structure of *Cg*AKR-1 revealed a conserved structure, known as the (β/α)_8_ barrel, within the AKR superfamily [46]. This structure consisted of eight β-strands in the central region surrounded by eight α-helices (see Additional file 1: Figure S6). The NADP-binding site is buried in the protein structure and is highly conserved throughout members of the AKR superfamily, despite the low similarity of other regions of the protein [47]. AKRs from family 1 generally show specificity for NADPH, corroborating our results [28]. *Cg*AKR-1 displays structural determinants that underlie the preference for NADPH, which is related to the positively charged arginine residues that bind to pyrophosphate backbone and the 5′ phosphate group of NADPH (Fig. 5) [48]. The amino acids W24, N47, Y52, H114, S166, N167, Y214, I217, S219, K276, S277, R282, E285, and N286 surrounded the cofactor (Fig. 5a). In addition, the catalytic residues were identified as D47, Y52, K81, and H114 and were also conserved. In general, the amino acid composition of the substrate-binding sites in AKRs is diverse and confers different substrate specificities to each AKR subfamily [30]. The inner region of the substrate-binding site was found by the C-terminal region of the β-strands together with the NADP nicotinamide group, and the binding site entrance was composed by loops connecting β-strands with α-helices (Fig. 5b).

The superposition of *Cg*AKR-1 chain A with AKRs complexed with substrates indicated that the substrate-binding site was delimited by the amino acids W24, M51, Y52, H114, L115, W128, R229, F314, and M316 (Fig. 5c).

One major feature of the *Cg*AKR-1 structure was the presence of a longer loop (known as loop B) between the seventh and eighth β-strands of the barrel (residues L222 to L242) compared with that of other AKRs (Fig. 5b, d). This longer loop also exhibited high mobility based on high B-factor in chain A, which could not be modeled in chain B owing to the absence of a defined electron density. According to Barski et al. [30], loop B is part of a “hot spot” for variability between the AKR families and is responsible for multiplicity of substrate specificity and kinetic properties. In addition, loop B from AKR1A and AKR1B has an open-and-close movement for cofactor entrapment [49]. Consequently, the unparalleled long loop B from *Cg*AKR-1 seemed to play an important role, not only in cofactor binding but also in substrate interaction owing to the arginine (R229) positioned towards the substrate-binding site (Fig. 5).

### *Cg*AKR-1 exhibited efficient hemicellulosic hydrolysate detoxification and improved yeast conversion of xylose to ethanol

**Fig. 6:**
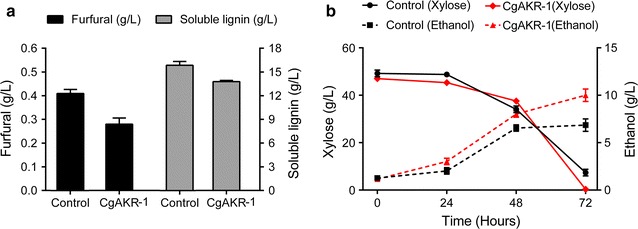
Effects of *Cg*AKR-1 on hemicellulosic hydrolysate detoxification followed by alcoholic fermentation using *S. stipitis*. The fermentation was carried out at 30 °C and 200 rpm for 72 h. Analysis was carried out by HPLC with three biological replicates. **a** Amounts of furfural and soluble lignin after detoxification; **b** ethanol production by *S. stipitis* and xylose consumption by *S. stipitis*

*Cg*AKR-1 was added to the hemicellulosic hydrolysate prior to fermentation in order to validate the detoxification capacity of the enzyme. After the enzymatic detoxification step, 32% of furfural and 15% of soluble lignin were eliminated (Fig. 6a). Fermentation of the detoxified hemicellulosic hydrolysate by *Scheffersomyces stipitis* at over 72 h was also evaluated (Table 2). There was a 45% increase in ethanol production compared with that of the control (control: 5.59 g/L; *Cg*AKR-1: 8.84 g/L; Fig. 6b). The concentration of cells after fermentation was not significantly altered (control: 19.5 ± 1.3 g/L; *Cg*AKR-1: 18.4 ± 1.5 g/L).

**Table 2:** *S. stipitis* fermentation parameters after hemicellulosic hydrolysate detoxification by *Cg*AKR-1

Parameters	Control experiment	Detoxification by *Cg*AKR-1
Yp/s (g/g)	0.13	0.19
Y (%)	26.19	37.12
Xylose consumption (%)	85.00	99.28

*Yp/s* xylose conversion factor in ethanol, *Y* percentage of ethanol production

According to Wahlbom [50], fermentation inhibitors can cause the depletion of redox cofactors during fermentation, resulting in slower, decreased ethanol production by yeast. The inhibitory effects can vary according to the chemical functional groups, and aldehydes are more harmful than other functional groups [11]. The diversity of inhibitory aldehyde structures reduced by *Cg*AKR-1 indicated that this enzyme was able to enhance alcoholic fermentation of this type of biomass by the detoxification of both furfural and phenolic and aldehyde derivatives from lignin (Table 1).

The detoxification of hemicellulosic hydrolysates is of biotechnological interest, and many detoxification methods have been reported in the literature [9, 11, 14, 17, 32, 51]. Laccases and peroxidases are being applied in the development of enzymatic cocktails for detoxification of lignin components, consuming or generating ROS such as H_2_O_2_ to remove soluble lignin in fermentation medium; thus, PAD enzymes such as *Cg*AKR-1 could also be applied for this purpose [10, 52, 53]. The degradation of lignin and cellulose, which are covalently linked in the biomass (through hemicelluloses), could be performed by oxidative steps [54]. Thus, we suggested that the hydrogen peroxide produced by *Cg*AKR-1 could oxidize the soluble lignin as well [55].

### *Cg*AKR-1 improved lignocellulose hydrolysis by H_2_O_2_ production

**Fig. 7:**
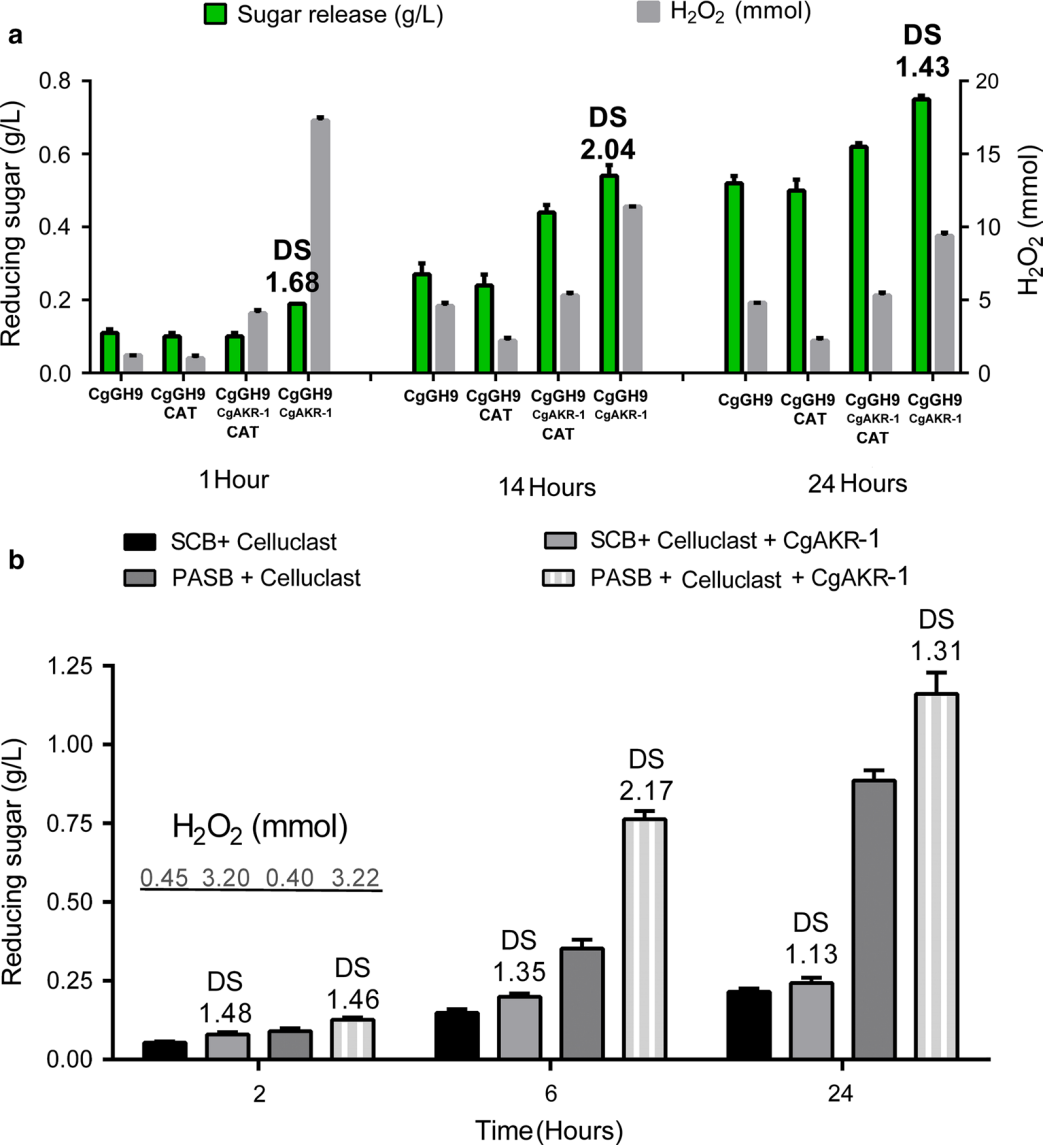
Effects of H_2_O_2_ generation during BG hydrolysis and hydrolytic synergy of enzymatic cocktails and *Cg*AKR-1. **a** One hundred nanograms of each enzyme (*Cg*GH9, *Cg*AKR-1, and a commercial catalase), NADPH, and 1.0% BG were used in the assays (Mix). The reaction was carried out at 30 °C for 1, 14, or 24 h. The DS was calculated as follows: (g/L) of reducing sugar of (*Cg*GH9 + enzyme)/(g/L) of reducing sugars of *Cg*GH9. No hydrolysis was detected by *Cg*AKR-1 in the absence of *Cg*GH9. **b** Celluclast was used for hydrolysis at 10 FPU/g of raw sugarcane bagasse (SCB) or phosphoric acid-pretreated sugarcane bagasse (PASB) and (0.5 mg of *Cg*AKR-1 plus NADPH)/g sugarcane bagasse. The reactions were performed at pH 5.7 for 2, 6, or 24 h at 30 °C and 1000 rpm. The degree of synergism (DS) was calculated as follows: g/L released sugar (Celluclast plus *Cg*AKR-1)/g/L released sugar (Celluclast). Denatured *Cg*AKR-1 was used as the control for all substrates. No hydrolysis was detected by *Cg*AKR-1/NAPDH in the absence of Celluclast

Next, we performed assays combining *Cg*AKR-1, *Cg*GH9 (*C. gestroi* endoglucanase [56]), and CAT (a commercial catalase that catalyzes the decomposition of H_2_O_2_ to water and oxygen) in order to evaluate whether the synergism of these enzymes on barley beta-glucan saccharification could be correlated with ROS generation. Hydrolysis with *Cg*GH9 released reducing sugars and background H_2_O_2_ production (Fig. 7). The addition of *Cg*AKR-1 to *Cg*GH9 improved the hydrolysis of beta-glucan, with a degree of synergism (DS) of 1.68, generating 17 mmol of H_2_O_2_ after a 1-h reaction. The maximum cooperation between the enzymes was found after 14 h of hydrolysis (DS: 2.04). However, the addition of a CAT enzyme to this reaction abolished the synergism and concomitantly led to lower production of H_2_O_2_ (Fig. 7a).

There were synergistic enzymatic interactions between *Cg*AKR-1 and *C. gestroi* endoglucanase. Our data indicated that the additive enzymatic activity could be mediated by ROS because improvement of glucan polysaccharide hydrolysis was correlated with H_2_O_2_ production (Fig. 7a). Glucan oxidation occurs via H_2_O_2_ through generation of new carbonyl and carboxyl groups in the polysaccharide, which could cleave the glucosidic bonds of cellulose [42, 43]. Several enzymes have been reported to improve lignocellulose hydrolysis in the presence of H_2_O_2_ and other oxygen species [45, 57–60].

Moreover, to further explore the potential biotechnological applications of this enzyme, we performed Celluclast 1.5 L supplementation with *Cg*AKR-1, which resulted in significant improvements in saccharification yield (Fig. 7b). The highest DS values were found for the hydrolysis of phosphoric acid-pretreated sugarcane bagasse (PASB; 1.48 at 2 h, 2.17 at 6 h, and 1.31 at 24 h; Fig. 7b). In contrast, *Cg*AKR-1 synergistically enhanced the hydrolysis of raw sugarcane bagasse (SCB) at early time points (DS of 1.46 at 2 h), with reduced effects at later time points (DS of 1.13 at 24 h). In both cases, H_2_O_2_ generation was found to correlate with improved saccharification (Fig. 7b).

The major difference in composition between SCB and PASB is regarding the content of hemicelluloses and lignin (PASB composition, as a percentage of dry mass: 59.0% cellulose, 1.8% hemicellulose, and 30.0% lignin [61]; SCB composition as a percentage of dry mass: 42.8% cellulose, 25.8% hemicellulose, and 22.1% lignin [62]). Lignin is responsible for blocking cellulolytic enzymes acting in bagasse fibers by nonproductive binding [11]. Hence, improvement of commercial cocktail performance is related to the generation of H_2_O_2_ by *Cg*AKR-1, which could result in lignin degradation through the cleavage of lignin–carbohydrate linkages, such as β-1,4 aryl ether linkages, and loss of cellulose crystallinity [63].

Collectively, our data suggested that oxidative cleavage mechanisms could significantly improve the yields of plant biomass deconstruction to biomass, demonstrating potential bioproduct applications [64]. Classical cellulase mixtures (e.g., Celluclast) do not generate reasonable yields of H_2_O_2_ or ^·^OH radical during reaction with biomass (see Additional file 1: Figure S8). These redox agents, which are active against lignocellulose, can be generated by PAD enzymes, which are therefore good candidates for the formulation of next-generation lignocellulosic cocktails (see Additional file 1).

According to the data presented in this study, *Cg*AKR-1 improved lignocellulose saccharification and yeast fermentation via two different proposed mechanisms: 1) the reduction of fermentation inhibitory compounds found in lignocellulose, and 2) the promotion of synergistic enzymatic interactions with glycoside hydrolases. However, there were some limitations to this study. First, the saccharification process at 50 °C is limited by the low thermal stability of *Cg*AKR-1. Additionally, we used a host that would not be suitable for industrial expression. Thus, improvement of thermostability through protein engineering and protein production by filamentous fungi could facilitate the industrial application of this method using endogenous enzymes from termites as a potential tool for biomass conversion. Therefore, studies involving *Cg*AKR-1 have greatly improved our understanding of termite biology and the role of this protein in both saccharification and fermentation steps as a “multipurpose enzyme,” functioning to mediate process integration during second-generation ethanol production and for green chemistry purposes.

## Conclusion

This work describes a founding member of AKR superfamily 1I, providing a basis for the involvement of endogenous enzymes, as components of the *C. gestroi* digestome, in redox mechanisms. Biotechnologically, *Cg*AKR-1 was found to be a versatile enzyme that was capable of detoxifying hemicellulosic hydrolysates for pentose fermentation and enhancing SCB saccharification via glycoside hydrolases. *Cg*AKR-1 provided a basis for the development and application of integrative and multipurpose enzymes as components in the bioethanol and biochemical production chain.

## Methods

### Collection and treatment of *C. gestroi*

For gene amplification and gut isolation, specimens of *C. gestroi* were maintained in the Termite Laboratory of the Biology Department, UNESP, Rio Claro, São Paulo, Brazil (22° 23′S, 47° 31′W) after collecting them from field colonies with traps of corrugated cardboard. Termites were kept at 25 ± 2 °C and fed on pinewood with 10% humidity until use.

### Phylogenetic tree of the AKR superfamily

The amino acid sequence (GenBank accession number: KU686221) of *C. gestroi* [26] was evaluated by comparison with multiple sequence analysis in the PFAM database, indicating the presence of a conserved domain from the AKR superfamily. To elucidate the function of this AKR, the amino acid sequence identified as *Cg*AKR-1 was submitted to phylogenetic analysis [35]. The phylogenetic tree was generated as described previously, including specifications according to the AKR superfamily homepage [35]. Briefly, the superfamily tree was constructed with the neighbor-joining method using 1000 bootstraps [65]. The tree was drawn to scale, with branch lengths in the same units as those of the evolutionary distances used to infer the phylogenetic tree. The evolutionary distances were computed using the JTT matrix-based method [66] and are presented as the number of amino acid substitutions per site. Previous analyses including all AKR family members were performed, confirming the classification of *Cg*AKR-1 in the AKR-1 family [35], as presented in Additional file 1: Figure S1. Subsequently, a refined analysis of 53 amino acid sequences, including known AKRs from family 1, such as *Cg*AKR-1 and related and well-described AKRs, was performed. All positions with less than 95% site coverage were eliminated. There were a total of 307 positions in the final dataset. All the analyses were conducted in MEGA6 [67].

### Cloning and expression of *C. gestroi* AKR

The sequence encoding full-length *CGAKR1* was amplified from *C. gestroi* cDNA using a standard polymerase chain reaction (PCR) method, as previously described [56]. Two nucleotide primers were used, as follows: (forward, 5′-TAAAATGCTAGCATGCCTAAACAACTGAGCAGT-3′, and reverse, 5′-TATTATGGATCCCTAATAAGGCTCATCATACGGGT-3′; restriction enzyme recognition sites are underlined). The PCR product was recovered after 1% agarose electrophoresis and further digested with *Nhe*I and *Bam*HI enzymes according to the manufacturer’s instructions. Finally, the double-digested PCR product was ligated into the pET-28(a) vector (Novagen) after treatment with the same two enzymes, allowing for insertion of a 6X-His tag sequence at the N-terminal position [56]. After cloning and sequencing, gene characterization was performed using Protparam, SignalP, and SecretomeP platforms. The *E. coli* strain ArcticExpress (DE3-T7 promoter; Agilent Technologies) competent cells were transformed with the pET-28a (Novagen)/*CGAKR1* plasmid and plated in selective solid LB medium containing kanamycin (50 mg/L). Cells from a single colony were grown in liquid LB containing kanamycin 50 mg/L for 16 h at 37 °C and 200 rpm. The cultures were then diluted in 600 mL fresh LB medium containing kanamycin and grown at 30 °C and 200 rpm for 4 h. The temperature and rotation were then reduced to 12 °C and 120 rpm, respectively. After 1 h of acclimation, expression of the recombinant protein was induced by the addition of 1 mM/L isopropyl β-d-1-thiogalactopyranoside. After 24 h, the cells were harvested by centrifugation at 8500×*g*. The cells were resuspended in lysis buffer (20 mM sodium phosphate [pH 7.5], 500 mM/L NaCl, 5 mM imidazole, 80 g/L egg lysozyme, and 5 mM polymethylsulfonyl fluoride [PMSF]) and then disrupted in an ice bath using an ultrasonic processor (seven pulses of 10 s at 500 W; VC750 Ultrasonic Processor, Sonics Vibracell). After that, the AKR from the supernatant was purified by chromatography using an AKTA FPLC system (GE Healthcare, Waukesha, WI, USA) using a 5-mL HiTrap Chelating HP column (GE Healthcare) charged with Ni^2+^ followed by a Superdex 200 10/300 GL column (GE Healthcare), as previously described [68]. The concentration of purified AKR was measured using a NanoDrop 2000c instrument (Thermo Scientific, USA) and calculated using the molar extinction coefficient (37,025^1^ cm^−1^).

### Crystallization, X-ray diffraction, and structure determination

The purified protein was concentrated to 10 mg/mL prior to crystallization assays. Initial crystallization experiments were set up using the sitting drop vapor diffusion method on a Honey Bee 963 robot at the ROBOLAB facility (LNBio-CNPEM) in a 96-well plate with drops composed of 0.5 µL protein solution plus 0.5 µL reservoir solution. Commercial kits from Hampton were used as initial conditions. A second round of crystallization was performed to refine the hits obtained in first experiment using the hanging drop vapor diffusion technique with the drops composed of 2 µL of protein solution and 2 µL of reservoir solution. All crystals were grown at 18 °C. The X-ray diffraction data were collected at 100 K using a beamline MX-2 with a Brazilian Synchrotron Light Source–LNLS (Campinas, Brazil), equipped with a Pilatus 6 M detector. Once the crystals were dissolved in the presence a cryoprotectant, they were directly transferred from the drops to the goniometer. The spots located in the ice ring areas were excluded for data indexing and integration. The collected data were indexed and integrated with XDS [69] and scaled with Aimless [70]. The initial phases were calculated by molecular replacement with Phaser [71] using the structure of aldose reductase from *Schistosoma japonicum* (PDBid 4hbk) as a search model. The structure adjustment and analysis were made with COOT [72], and refinement was performed with Phenix [73]. The final structure factors and model were validated with Molprobity [74] and deposited in the PDB databank with accession code 5KET. For more crystallization and data processing details, please see the Additional file 1: Table S1 and Figure S6.

### *Cg*AKR-1 immunolocalization in *C. gestroi* gut tissue

The immunolocalization of *Cg*AKR-1 was analyzed in the termite gut according to methods described by Price et al. [75]. The purified protein *Cg*AKR-1 was used to produce polyclonal antibodies in rabbits (commissioned with RHEABIOTECH Ltd.), according to standard protocols (www.rheabiotech.com.br). IgG fractions were purified from rabbit serum by protein G affinity chromatography (Amersham), according to the manufacturer’s instructions. Eluted antibodies were concentrated to 10 mg/mL.

For analysis of protein immunolocalization, termites were washed in 70% (v/v) ethanol, followed by phosphate-buffered saline (PBS), and the complete guts were then dissected out in PBS with PMSF (0.2 mM), ethylenediaminetetraacetic acid (EDTA; 1 mM), and leupeptin (20 μM). After dissection, guts were transferred to a tube containing 2% (w/v) paraformaldehyde. The gut tissue was then fixed for 2 h at room temperature. After fixation, gut tissues were washed several times in 1× PBS. Nonspecific antibody binding was prevented by incubating the gut tissue for 1 h in a solution containing 4% (v/v) Triton X-100 with 2% (w/v) bovine serum albumin (BSA) in PBS. After blocking, the tissue was incubated in primary antibody with shaking at 4 °C for 24 h. Anti-*Cg*AKR-1 antibodies were used at a concentration of 1:1000 in antiserum buffer (0.4% [v/v] Triton X-100 with 2% [w/v] BSA in PBS). Gut tissues were then washed in PBS at 4 °C for 24 h. AlexaFluor 568 (red) secondary antibodies were incubated with the tissue specimens at a concentration of 1:200 in antiserum buffer at 4 °C for 18 h. The secondary antibody solution was removed, and the tissues were washed in PBS at 4 °C for 18 h. Immunostained gut samples were mounted on glass slides. Control experiments were run in parallel and consisted of a primary antibody-only control and a secondary antibody-only control. Control experiments were set up following the same procedure, except the appropriate antibody incubation stage was omitted (see Additional file 1: Figure S4). Additionally, the immunolocalization of termite endoglucanase (*Cg*GH9) was analyzed as a positive control (see Additional file 1: Figure S5). Additional information about equipment and settings can be found in Additional file 1.

### Enzymatic assay

AKR activity was assayed spectrophotometrically at 30 °C by monitoring the decrease in the absorbance of NADPH at 340 nm in microplates. The standard assay mixture (0.2 mL) was composed of 50 mM sodium phosphate buffer (pH 7.0), 5.0 mmol/L substrate (2-nitrobenzaldehyde, furfural, HMF, etc.), and 2.5 µg *Cg*AKR-1. Additionally, 0.2 mmol/L NADPH was added to the plate to initiate the reaction, and the reaction rate was measured against an identical blank with no enzyme added. Activity was measured for 5 min. One unit of enzyme activity was defined as the amount of enzyme catalyzing the oxidation of 1 µmol NADPH per minute (μmol/mg/min) under the described assay conditions. The kinetic parameters of the purified enzyme were determined by assaying activities at different NADPH concentrations using 5 mM 2-nitrobenzaldehyde as the substrate. Furthermore, activities at different 2-nitrobenzaldehyde concentrations were measured using 0.2 mM NADPH. Optimal pH was determined using phosphate buffer (pH 3.0–8.0) and Tris buffer (pH 8.5–9.0) at 100 mM at 30 °C. Thermo residual stability was measured after enzyme incubation for 30 min at different temperatures at a fixed pH of 5.7. The results were expressed in relative activity (%).

### Detection of ROS

H_2_O_2_ generation was measured using Amplex Red, as previously described [76], with a Catalase Assay Kit (cat. no. A22180; Life Technologies) at 571 nm. Fifty microliters of the sample was used in the assay. A hydroxyphenyl fluorescein (HPF) assay kit was used for ^·^OH detection (see Additional file 1), and samples were incubated with 40 µL of 50 µM HPF [77]. Denatured *Cg*AKR-1 was used as a negative control, and one reaction with 5 mM FeSO_4_ was performed to evaluate the Fenton chemistry if H_2_O_2_ was being generated by *Cg*AKR-1. The reactions were kinetically monitored over 30 min at 35 °C using a plate reader fluorometer (Molecular Devices). The excitation wavelength was 488 nm, and the emission wavelength was 515 nm. All reactions were performed in triplicate.

### Detoxification of hemicellulosic hydrolysate by *Cg*AKR-1

The detoxification step was performed by *Cg*AKR-1 application in the liquid fraction (hemicellulosic hydrolysate) obtained after pretreatment. This fraction consisted of monomeric sugars, organic acids, furanic aldehydes, and phenolic compounds, with the following chemical composition: arabinose (21.59 g/L), glucose (31.90 g/L), xylose (209.21 g/L), cellobiose (5.08 g/L), formic acid (0.66 g/L), acetic acid (6.45 g/L), HMF (0.12 g/L), furfural (0.85 g/L), levulinic acid (1.12 g/L), and soluble lignin (25.32 g/L), as reported by Santoro et al. [78]. To evaluate whether *Cg*AKR-1 could be applied to enzymatic in situ detoxification of fermentation inhibitors in hemicellulosic hydrolysate, samples were sterilized at 111 °C for 5 min and diluted twice with 100 mM potassium phosphate buffer (pH 5.0, adjusted with 6 M NaOH, which was added slowly to ensure that the lignin would not precipitate). Subsequently, 1 mg *Cg*AKR-1 and NADPH (2 µM) were added to the reaction and incubated at 30 °C for 16 h. After incubation, hemicellulosic hydrolysate alcoholic fermentation was performed. The yeast used in this study was the wild-type *S. stipitis* NRRL Y7124 strain, a xylose-fermenting microorganism capable to ferment the hemicellulosic hydrolysate [79, 80]. During this process, 3 g/L yeast extract was added to the liquor, and *S. stipitis* yeast was inoculated at a final concentration of 10 g/L in 50 mL; the hemicellulosic hydrolysate was present at a final dilution of 4× (+-xylose, 50 g/L). Thereafter, the experiment was incubated at 30 °C with continuous agitation at 200 rpm for 72 h, as described by Dussán et al. [81], using shake flasks. Glucose, xylose, acetic acid, glycerol, xylitol, furfural, and ethanol were measured during the fermentation according to the analytical measurements described below. Yeast concentrations (g/L) and kinetic parameter calculations (Yp/s: xylose conversion factor in ethanol; Y: percentage of ethanol production) were measured and calculated as described by Santos et al. [82].

### Analytical measurements

The DNS method [83] was used for measurement of total reducing sugars. Analysis of xylose, furfural, and ethanol was performed using high-performance liquid chromatography as described by Rocha et al. [84]. Sugars, organic acids and fermentation products were quantified by Aminex HPX 87H (300 7.8 mm; Bio-Rad, Hercules, CA, USA) at 35 °C using 5 mM H_2_SO_4_ as the mobile phase at a flow rate of 0.6 mL/min. For the analysis of furfural and HMF, a reversed-phase HPLC equipped with an Acclaim 120 C18 column (150 mm × 4.6 mm, 3 μm) and a single wavelength UV detector were used. The mobile phase was water–acetonitrile 1:8 (v/v) with 1% acetic acid (v/v) at a flow rate of 0.8 mL/min. For greater accuracy and specificity, the samples were filtered through a Millex 22-µm PVDF filter, and the filtrate was injected into the HPLC system.

SCB and PASB were characterized according to the methods of Sluiter et al. [85]. The lignocellulosic materials were air-dried to less than 10% (w/w) of moisture content and then milled to obtain particle sizes of 0.12 mm in a knife mill (Pulverisette 19; Fritsch GmbH, Idar-Oberstein, Germany). After quantification of extracts (performed only for the raw bagasse) and ashes, the materials were milled again in a shear and impact mill (Pulverisette 14; Fritsch GmbH), yielding a particle size of less than 0.5 mm; these particles were used for analysis of structural carbohydrates (glucan, xylan, and arabinan) and soluble and insoluble lignin. The moisture content of the biomass was determined using an automatic infrared moisture analyzer (MA35; Sartorius Gmbh, Goettingen, Germany).

All analyses in this work were performed in triplicate, and Student’s *t* tests were performed. Results were considered significant when the *P* value was less than 0.05.

### Enzyme synergism and the role of ROS during glucan hydrolysis

To evaluate *Cg*AKR-1 synergism with glycoside hydrolases and the relationship with H_2_O_2_ generation, 1.0% BG was hydrolyzed in 100 mM phosphate buffer (pH 5.7) in a total volume of 500 µL at 30 °C and 1000 rpm for 24 h. Samples were collected at 1, 14, and 24 h, and 100 ng of each enzyme (*Cg*AKR-1 and *Cg*GH9) and 200 nmol/L NADPH were combined as the “Mix”. A commercial catalase (H_2_O_2_ decomposer; cat. no. A22180; 1 U/mL; Life Technologies) was used to evaluate the effects of ROS inhibitors on the DS. After hydrolysis, the H_2_O_2_ generation of each sample was measured with Amplex using 50 µL of each reaction, and the total sugars were measured with the remaining sample. The analyses were performed in triplicate. No hydrolysis was detected by *Cg*AKR-1 or NAPDH in the absence of endoglucanase. The DS for sugar release was calculated as follows: (g/L) of reducing sugar of (*Cg*GH9 + enzyme)/(g/L) of reducing sugars of *Cg*GH9, as described by Goldbeck [86].

### SCB hydrolysis and composition

The SCBs were subjected to enzymatic saccharification with a commercially available enzyme preparation (Celluclast 1.5 L; Novozymes) at 10 FPU/g SCB and in combination with 0.5 mg *Cg*AKR-1 plus 6.25 mmol NAPDH/g SCB. The enzymatic hydrolysis was performed with 2% (w/v) SCB in 100 mM phosphate buffer (pH 5.7) at 30 °C. The reactions were carried out in 2-mL Eppendorf tubes using a Thermomixer microplate incubator (Eppendorf, Germany). Samples were centrifuged at 10,000×*g* for 15 min (5418 Centrifuge; Eppendorf) and filtered (Sepak C18; Waters). The SCB was provided by Usina da Pedra (Serrana-SP). The material was collected in the 2013/14 crop and was mechanically harvested after the final milling before juice extraction. The phosphoric acid pretreatment (PASB) was described in detail in previous studies [61]. Denatured *Cg*AKR-1 was used in all reactions lacking the enzyme. No hydrolysis was detected by *Cg*AKR-1 or NAPDH in the absence of Celluclast. The analyses were performed in triplicate. The DS for sugar release was calculated as follows: (g/L) of reducing sugar of (Celluclast + *Cg*AKR-1)/(g/L) of reducing sugars of Celluclast, as described by Goldbeck [85].

## Additional file

**Additional file 1.** Additional Figures S1–S7 and Table S1.

## Abbreviations

*C. gestroi*: *Coptotermes gestroi*
*Cg*AKR-1: *Coptotermes gestroi* aldo–keto reductase 1
*S. stipitis*: *Scheffersomyces stipitis*
H_2_O_2_: hydrogen peroxide
HMF: hydroxymethylfurfural
PAD: pro-oxidant, antioxidant, and detoxification enzyme
ROS: reactive oxygen species
HPF: peroxynitrite sensor
NADPH: reduced form of nicotinamide adenine dinucleotide phosphate
·OH: hydroxyl radical
*Cg*GH9: termite Endoglucanase *Cg*EG-1
BG: barley beta-glucan low viscosity
SCB: raw sugarcane bagasse
PASB: phosphoric acid-pretreated sugarcane bagasse
Mix: CgGH9 + CgAKR-1 + NADPH
