# Supplementary material for: The Coptotermes gestroi aldo–keto reductase: a multipurpose enzyme for biorefinery applications
Source: Biotechnol Biofuels. 2017 Jan 3;10:4. doi: 10.1186/s13068-016-0688-6 (PMC5209882; doi:10.1186/s13068-016-0688-6)
Supplement: Supplementary file 1 — Additional file 1. Additional Figures S1–S7 and Table S1. [file 13068_2016_688_MOESM1_ESM.pdf]

Supplementary Material

The Coptotermes gestroi aldo–keto reductase: a multipurpose enzyme for biorefinery applications

Robson Tramontina, João Paulo L. Franco Cairo, Marcelo V. Liberato, Fernanda Mandelli, Amanda Sousa, Samantha Santos, Sarita Cândida Rabelo, Bruna Campos, Jaciane Ienczak, Roberto Ruller, André R. L. Damásio and Fabio Marcio Squina

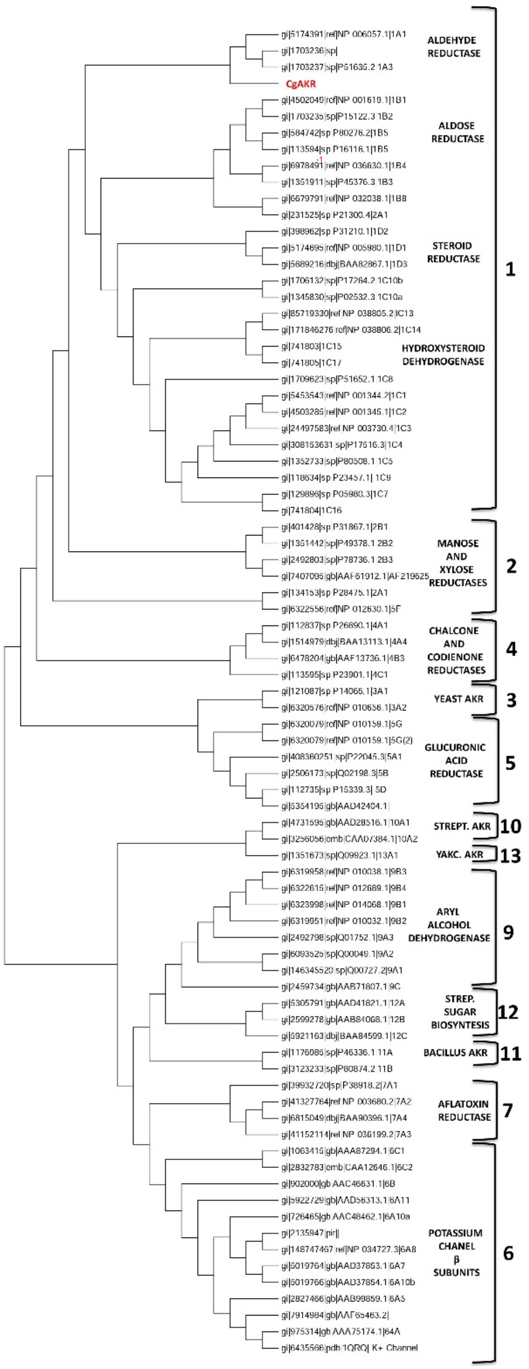

Supplementary Figure 1. AKR superfamily classification. The phylogenetic analysis was inferred using the neighbor-joining method [1]. The optimal tree with the sum of branchlength = 1823.28065972 is shown. The percentage of replicate trees in which the associated taxa clustered for bootstrap tests (500 replicates) [2]. The tree is drawn to scale, with branch lengths in the same units as those of the evolutionary distances used to infer the phylogenetic tree. The evolutionary distances were computed using a number of diferences method [3] and are in the units of the number of amino acid diferences per sequence. The analysis involved 81 amino acid sequences. All positions containing gaps and missing data were eliminated. There were a total of 148 positions in the final dataset. Evolutionary analyses were conducted in MEGA6 [4].

Heterologous production of CgAKR-1

SDS-PAGE was performed according to the method of Laemmli [5] using 12% gels.

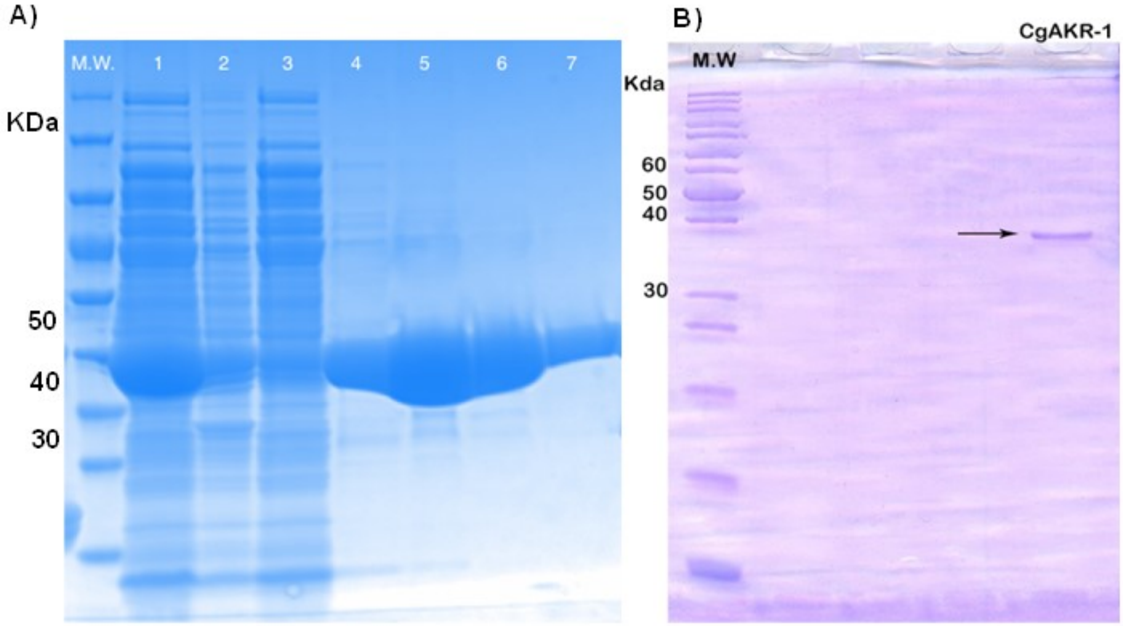

Supplementary Figure 2. Analysis of heterologous expression of CgAKR-1 in the Arctic Cell Expression System. CgAKR-1 was expressed at 12°C with shaking at 120 rpm for 24 h and purified using IMAC and GF. (A) IMAC purification with SDS-PAGE. Lanes: MW, molecular weight standard; 1, lysate; 2, pellet; 3, flowthrough; 4–7, IMAC fractions. (B) Gel filtration fraction. MW, molecular weight standard. The CgAKR-1 purified band is indicated by the arrow.

CgAKR-1 immunolocalization

Antibodies

Supplementary Figure 2 shows the results of anti-CgAKR-1 antibody analysis; the antibody showed affinity for the target, even at a dilution of 1:2000. The assay was performed by **RHEABIOTECH LTDA as described in the Materials and Methods section and the company's specifications.** The antibodies were analyzed using standard indirect enzyme-linked immunosorbent assays (ELISAs). The blocking step was carried out in phosphate-buffered saline (PBS; 137 mM NaCl, 2.7 mM KCl, 10 mM Na_2HPO_4 and 2 mM KH_2PO_4, pH 7.5) plus 2% BSA, and the detection was carried out with the following dilutions: 1:500, 1:1,000, 1:2,000, 1:4,000, 1:8,000, 1:16,000, 1:32,000, and 1:64,000. The revelation step was performed with a secondary antibody conjugated with peroxidase, and H_2O_2/OPD was used as the chromogen substrate. The reading was carried out at 492 nm. The purified IgG fractions were tested for specific binding to their targets by ELISA. The results showed that the anti-CgAKR-1 antibody could bind specifically with recombinant CgAKR-1 (Supplementary Figure 3).

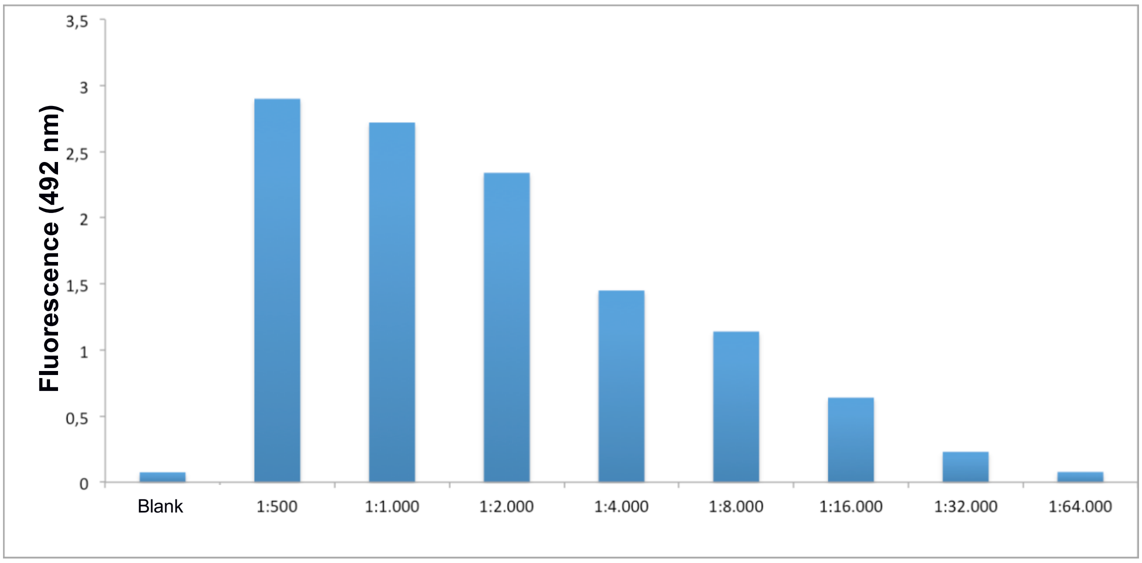

Supplementary Figure 3. Detection of CgAKR-1 by indirect ELISA using the polyclonal antibody.

The supplementary figure 3 shows the positive control which is the primary and secondary antibody incubations with the sample (1), the primary only and secondary antibody only negative controls (2 and 3 respectively). There was clear evidence of the antibody specificity for the protein CgAKR-1 in the sample (1A). Also, nonspecific fluorescence wasn't detected in the absence of primary only or secondary only antibody incubation with the sample.

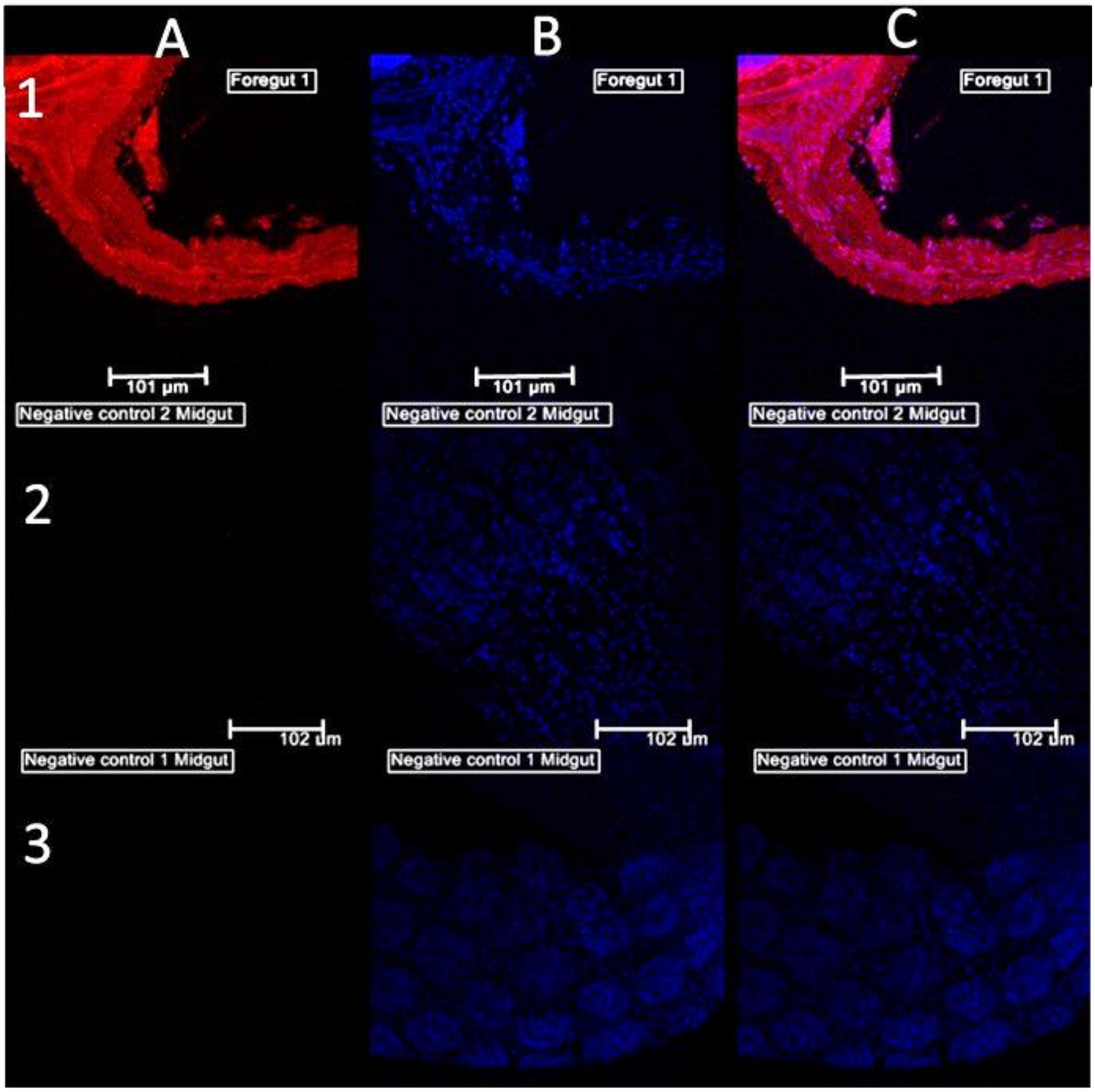

Supplementary Figure 4. Immunolocalization of CgAKR-1 in *C. gestroi* gut tissue. Gut tissues were incubated with primary anti-CgAKR-1 antibodies and fluorescent secondary antibodies (conjugated with AlexaFluor 568) and observed under a Leica DMI 6000 microscope. Red fluorescence represents CgAKR-1 immunolocalization in the foregut (1). Blue fluorescence represents the nuclei of all tissue cells, as determined using ProLong Antifade Reagents for Fixed Cells (B). (C) Represents the junction of images 1 and 2 (midgut). Similar experiments using either primary or secondary antibodies independently provided a control for autofluorescence and nonspecific binding of the fluorescent secondary antibody (2 and 3). Images from the red and blue channels were recorded independently and digitally overlaid to produce a final image.
As a positive control, termite endoglucanase was immunolocalized to the termite gut. The enzyme was secreted mainly in the foregut.

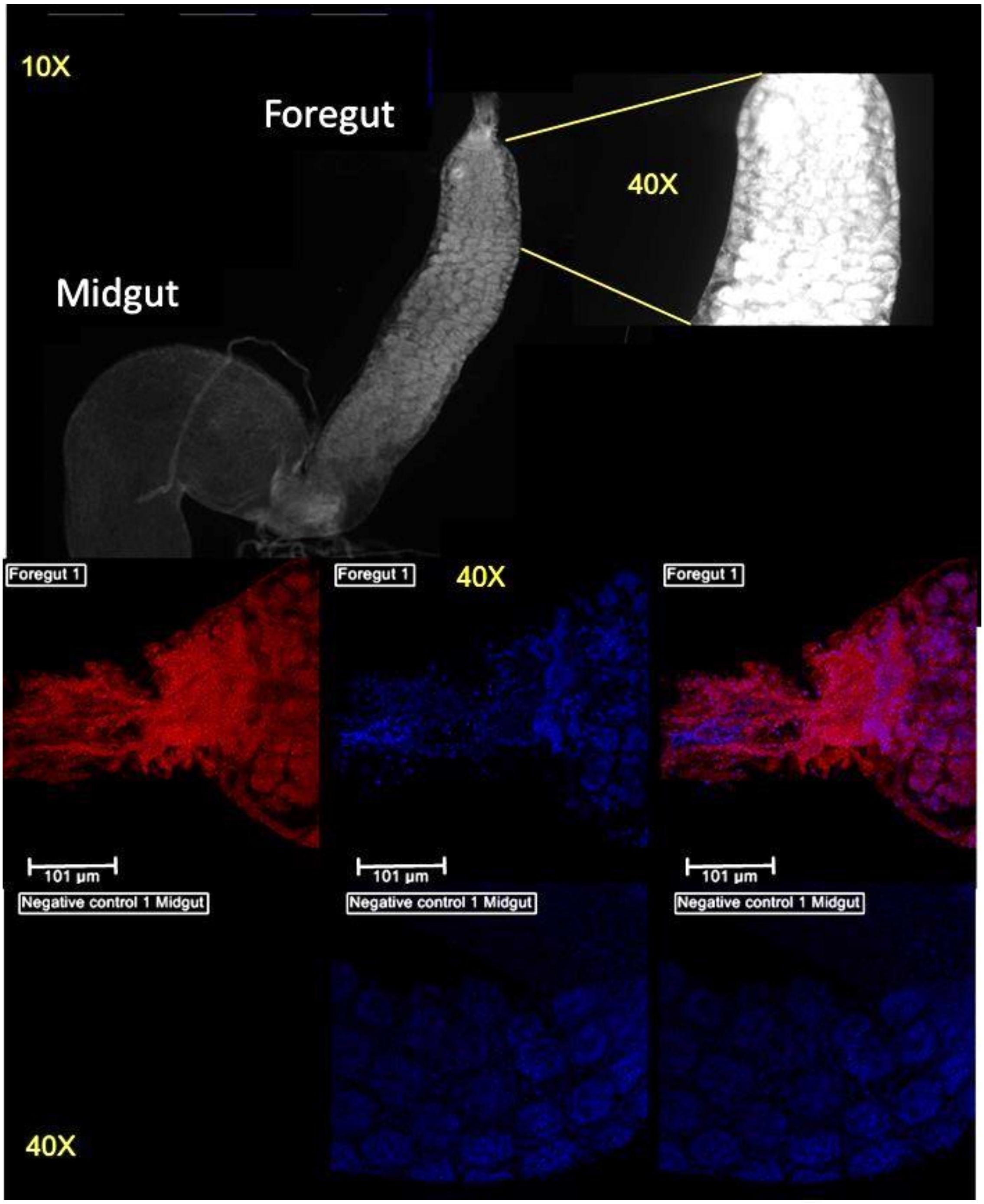

Supplementary Figure 5. Immunolocalization of CgGH9 in *C. gestroi* gut tissue. Gut tissues were incubated with primary anti-CgAKR-1 antibodies and fluorescent secondary antibody (conjugated with AlexaFluor 568) and observed under a Leica DMI 6000 microscope. Red fluorescence represents CgGH9 immunolocalization; blue fluorescence represents the nuclei of all tissue cells, as determined using ProLong Antifade Reagents for Fixed Cells. Similar experiments using either primary or secondary antibodies independently provided a control for autofluorescence and nonspecific binding of the fluorescent secondary antibody. Images from the red and blue channels were recorded independently and digitally overlaid to produce a final image.

Equipment and settings

The samples were examined at the Biological Imaging Facility of the Brazilian Biosciences National Laboratory (LNBio) at the Brazilian Center for Research in Energy and Materials (CNPEM) using a Leica TCS SP8 confocal microscope on a Leica DMI 6000 with 10× and 40× objectives. Images were collected using a 578 nm laser for excitation, with emission detection at 603 nm (AlexaFluor 568). The generated images were analyzed using Leica Application Suite Advanced Fluorescence (LAS AF) Version 4.3. The images were built through Microsoft® PowerPoint (2010).

CgAKR-1 Structural features

The first CgAKR-1 protein crystals were obtained under conditions containing 5% PEG400, 0.05 M magnesium sulfate, 2 M ammonium sulfate, and 0.1 M Tris base (pH 8.5). In order to obtain larger and well-formed crystals, manual screening was carried out by varying the initial condition. A crystal grown in 10% PEG400, 0.1 M magnesium sulfate, 2 M ammonium sulfate, and 0.1 M Tris base (pH 8.5) was diffracted, and a complete data set was collected at 2.85-Å resolution. The statistics are described in Supplementary Table 1.

Supplementary Table 1 - Statistics from X-ray diffraction data collection and refinement

	CgAKR-1
**Data collection**	
Wavelength (Å)	1.46
Space group	H32
Cell dimensions	
a, b, c (Å)	131.14, 131.14, 290.66
α, β, γ(°)	90, 90, 120
Resolution (Å)	44.74–2.85 (3.0–2.85)*
Total reflections	195360
Unique reflections	22740
R_{merge}	0.29 (1.65)
R_{pim}	0.10 (0.64)
I/σI	8.6 (1.5)
Completeness (%)	99.8 (99.9)
Redundancy	8.6 (7.5)
CC ½ (%)	98.7 (53.6)
**Refinement**	
R_{work} / $R_{free,}$ (%)	23.89/29.01
No. atoms	
Protein	4950
Ligand	96
B-factors	
Protein	50.6
Ligand	70.6
R.m.s. deviations	
Bond lengths (Å)	0.009
Bond angles (°)	0.968
Ramachandran	
Favored	95.25
Allowed	4.75
Outliers	0

* Highest resolution shell is shown in parentheses.

The structure of CgAKR-1 was solved by molecular replacement using an aldose reductase from *Schistosoma japonicum* (SjAR). Although CgAKR-1 has only 46.3% amino acid sequence identity with SjAR, the high structural similarity between them (RMSD = 0.61 Å) allowed molecular replacement for identification of the initial phases. The final crystallographic model was composed of two molecules in the asymmetric unit; however, there was no evidence that the enzyme formed a dimer in solution. Due

to poor resolution or absence of electron density, the following residues were not built in the final model: 1–6 from chain A; and 1–2, 129–136, 225–233, and 316–321 from chain B.

The two molecules in the asymmetric unit were very similar (RMSD = 0.32 Å), with the differences located in loops exposed to solvent. Both molecules were complexed with NADP, and, because the cofactor was not added in any step of expression, purification, or crystallization, the cofactor could have been supplied by the expression host (*Escherichia coli).* The presence of the cofactor with the enzyme, even after the purification steps, suggested a high affinity for the binding site. The high affinity of NADP for AKR members has been described previously [6].

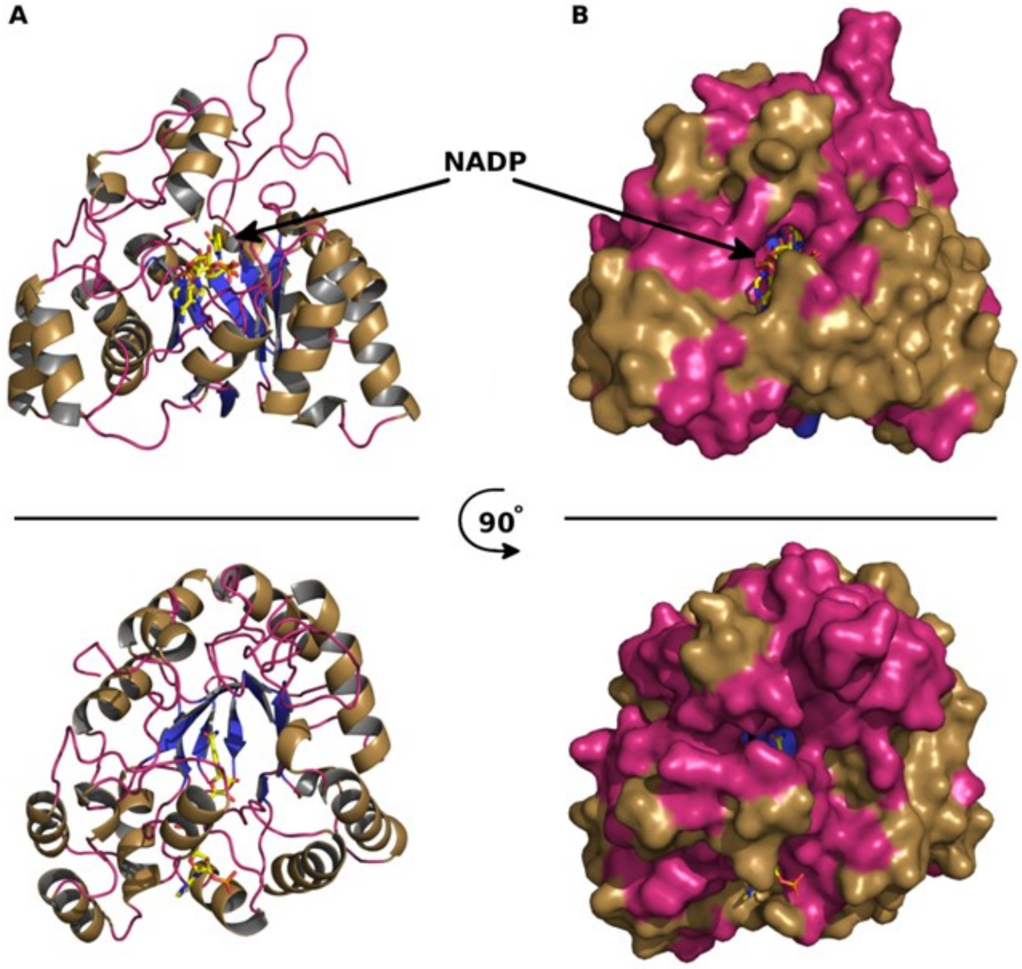

Supplementary Figure 6. Overall crystallographic structure model of CgAKR-1, chain A. (A) Cartoon representation showing conserved folding of AKR, known as the $(\beta/\alpha)_8$ barrel. The secondary structures are highlighted in different colors: β-strands in blue, α-helices in gold, and loops in pink. (B) Surface representation showing the NADP binding site crossing the protein structure.

Hydroxyl radical and peroxynitrite (HPF) assays for Fenton reaction detection

The capability of CgAKR-1 to generate H_2O_2 to enzymatically start the Fenton reaction was validated in assays using the $^{\bullet}OH$ radical and peroxynitrite sensor (HPF) (Supplementary Figure 6). As expected, no $^{\bullet}OH$ radicals were produced by CgAKR-1 alone. The •OH radical was produced via Fenton chemistry when Fe^{2+} was added to the reaction. As presented: $Fe^{2+} + H^2O^2 \rightarrow Fe^{3+} + {}^{\bullet}OH + OH-$.

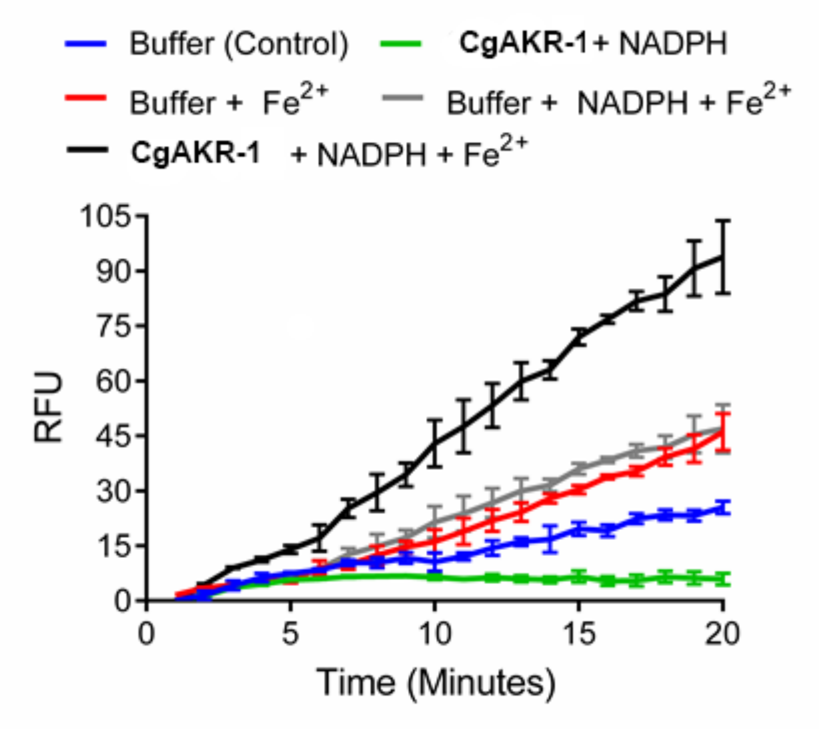

Supplementary Figure 7. $^{\bullet}OH$ generation by CgAKR-1, NADPH, and Fe^{2+}, as measured using an HPF assay kit (see the Materials and Methods), with fluorescence (RFU) measured using emission wavelengths of 515 nm for 20 min. All reactions were performed in triplicate in 100 mM phosphate buffer (pH 5.7) at 37°C.

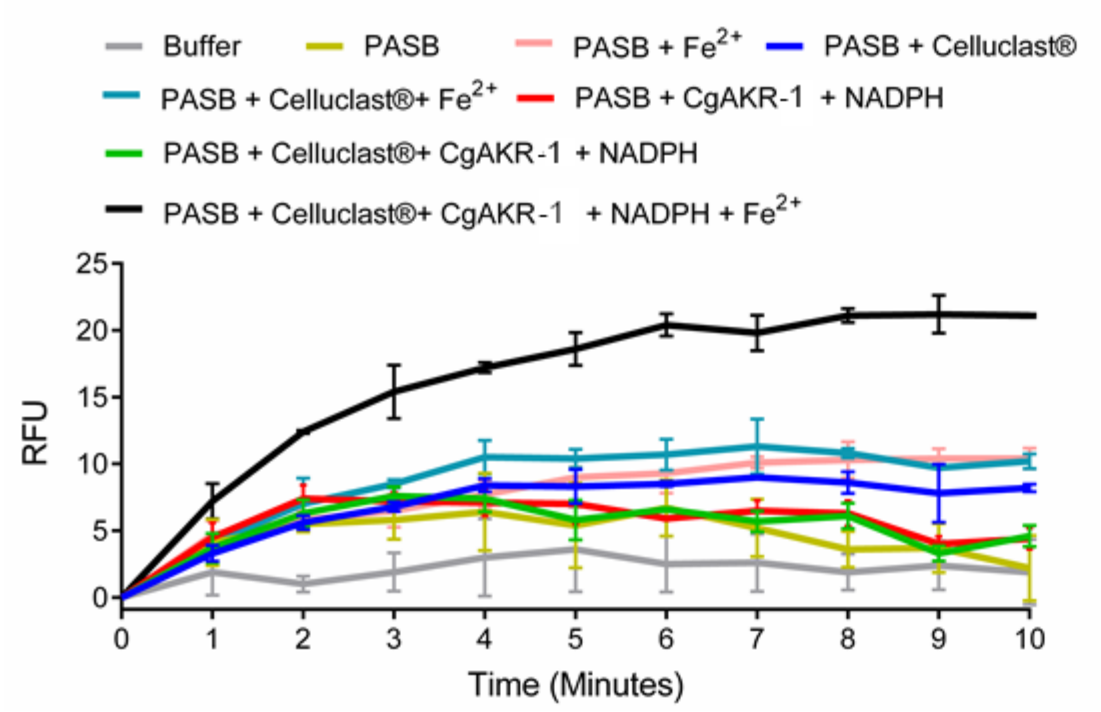

Supplementary Figure 8. •OH generation by CgAKR-1, NADPH, Celluclast, PASB, and Fe^{2+}, as measured using an HPF assay kit (see the Materials and Methods), with fluorescence (RFU) using emission wavelengths of 515 nm for 20 min. All reactions were performed in triplicate in 100 mM phosphate buffer (pH 5.7) at 37°C.

Supplementary references

1 Saitou N, Nei M. The neighbor-joining method: A new method for reconstructing phylogenetic trees. Mol Biol Evol. 1987;4:406-25.

2. Felsenstein J. Confidence limits on phylogenies: An approach using the bootstrap. Evolution. 1985;39:783-91.

3. Nei M, Kumar S. Molecular Evolution and Phylogenetics. Oxford University Press, New York, 2000.

4. Tamura K, Stecher G, Peterson D, Filipski A, Kumar S. MEGA6: Molecular Evolutionary Genetics Analysis version 6.0. Mol Biol Evol. 2013;30:2725-9.

5. UK Laemmli. Cleavage of structural proteins during the assembly of the head of bacteriophage T4. Nature. 1970;227:680-5.

6. Penning TM. The aldo-keto reductases (AKRs): overview. Chem Biol Interact. 2015;234:236-46.
